# Rhinovirus-induced Rapidly Progressing Acute Respiratory Distress Syndrome in an Immunocompetent Host

**DOI:** 10.7759/cureus.3997

**Published:** 2019-02-01

**Authors:** Sam Ngu, Sami Pervaiz, Akshay Avula, Michel Chalhoub

**Affiliations:** 1 Internal Medicine, Staten Island University Hospital, Staten Island, USA

**Keywords:** rhinovirus, acute respiratory distress syndrome (ards), immunocompetent host

## Abstract

A previously healthy, 59-year-old female presented with respiratory distress and dry cough for one week. Outpatient radiographic findings were suspicious for basilar pneumonia. Empiric broad-spectrum antibiotics were started; however, she continued to deteriorate rapidly over the next 48 hours, with chest X-ray showing diffuse bilateral multifocal airspace opacities consistent with acute respiratory distress syndrome. The ratio of partial pressure arterial oxygen to fraction of inspired oxygen was 225. She required a high-flow nasal cannula with a subsequent upgrade to the intensive care unit (ICU) for increasing respiratory compromise. Polymerase chain reaction (PCR) of the nasopharyngeal aspirate confirmed human rhinovirus (hRV). High-dose intravenous steroids were started as adjuvant therapy due to the rapid decline, presumably due to a dysregulated host immune response. After 10 days in the ICU, she was discharged with tiotropium and steroid taper. Historically thought to be limited to pandemic viruses, improved detection of hRV has led to its implication in serious respiratory disorders extending beyond the oropharynx in immunocompetent hosts. We report a rare case of hRV-induced severe acute respiratory distress syndrome (ARDS) in an immunocompetent host. This case highlights the need for the early identification of viral culprits, which can minimize the use of invasive diagnostic testing and antibiotic usage.

## Introduction

Human rhinoviruses (hRVs) are leading causes of upper respiratory tract infections worldwide [1]. Typically associated with the common cold, they are small, non-enveloped, single-stranded, positive-sense ribonucleic acid (RNA) viruses belonging to the family Picornaviridae and genus Enterovirus [2]. Three species of hRV (hRV-A, hRV-B, hRV-C) are now known to exist, with marked phylogenetic diversity such that immunity against any subtype is unlikely to confer a protection advantage against the others [2]. Historically, pandemic viruses, such as influenza viruses and coronaviruses, are known to produce serious lung injury and potentially lethal infections. Immunocompromised patients and those at extremes of age have a worse prognosis due to insufficient or immature immune response [3]. It can also increase the risk of respiratory complications in those with underlying asthma [4]. The improved detection of hRV has led to its implication in serious respiratory disorders extending beyond the oropharynx in immunocompetent adult hosts, including pneumonia and acute respiratory distress syndrome (ARDS).

hRVs can infect the human host via direct contact, contact with fomites, or in an airborne manner [2]. In the respiratory tract, it enters the cell via macropinocytosis and clathrin-dependent and clathrin-independent endocytosis [5]. Unchecked viral replication draws the recruitment of immune cells that release various cytokine profiles, which increases the permeability of the alveolar-capillary membrane in the absence of elevated hydrostatic pressure in the pulmonary veins [6]. The resulting injury to lung parenchyma, termed diffuse alveolar damage, leads to hypoxia, pulmonary edema, plasma protein leakage, and further macrophage and neutrophil infiltration. Histologically, hyaline membranes in the alveoli can be observed. During the inflammatory process, changes to surfactant composition and functionality result in alveolar collapse. The exact pathogenesis of virus-induced ARDS remains to be elucidated but may be related to a dysregulated host inflammatory response as opposed to viral-mediated injury [3].

The diagnosis of ARDS is based on the Berlin criteria. Clinical suspicion is required for any patient presenting with a respiratory viral infection, hypoxemia, and bilateral opacities on chest radiography [3]. The development of respiratory distress should occur within one week of viral antigen detection with minimal to absent contribution from cardiogenic pulmonary edema or volume overload states.

Rhinovirus-induced ARDS is extremely rare in immunocompetent adult hosts, with only three documented cases on PubMed to date. We report a case of a previously healthy 59-year-old female who presented with respiratory distress that rapidly progressed to ARDS.

## Case presentation

**Figure 1:**
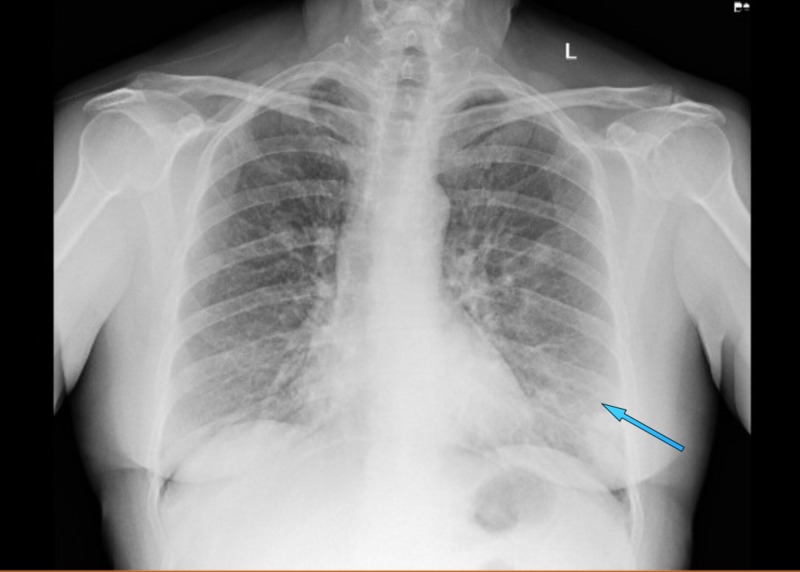
Initial anterior-posterior chest radiograph obtained on hospital admission showing a small left basilar airspace opacity (blue arrow)

**Figure 2:**
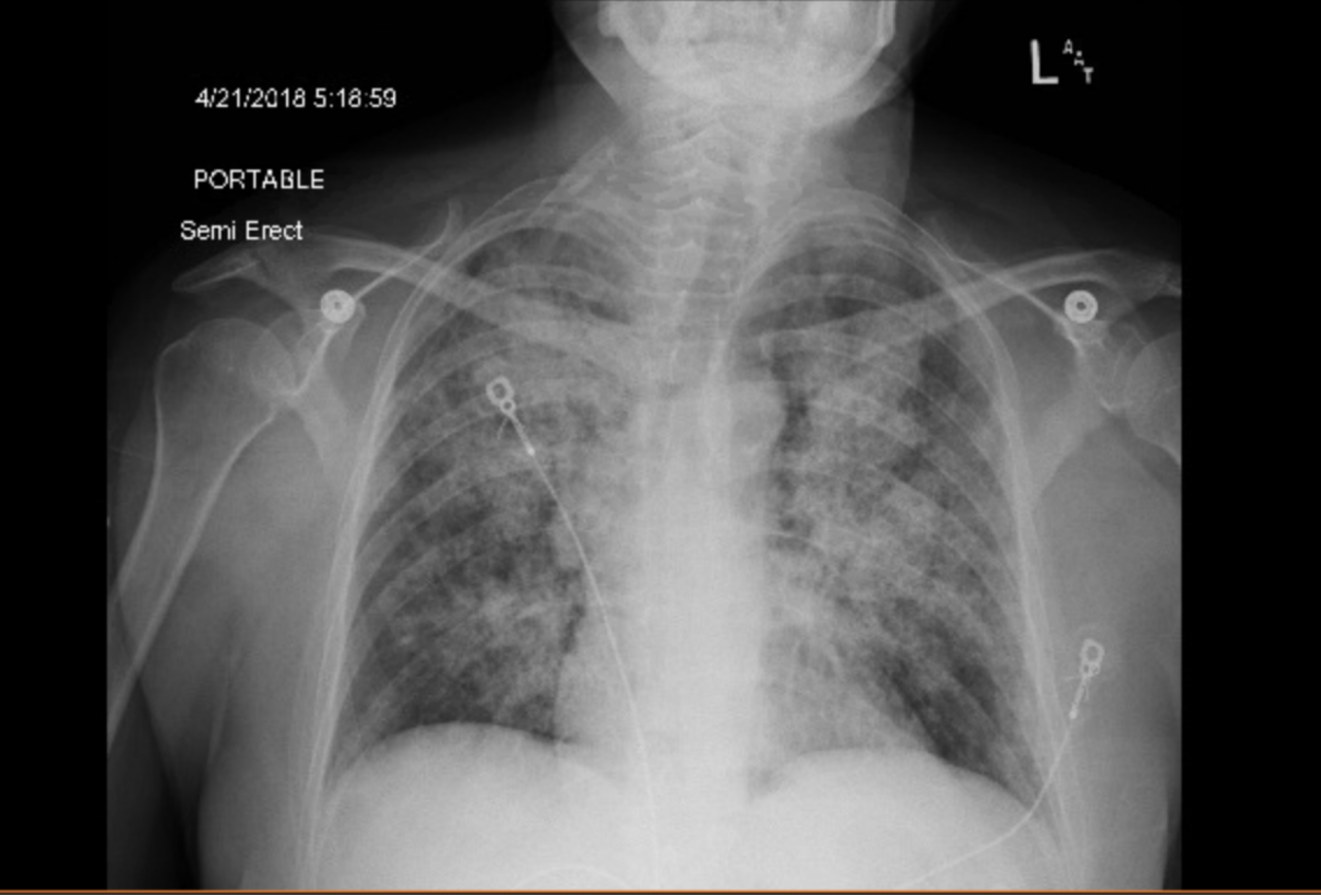
Repeat anterior-posterior chest X-ray obtained for increasing shortness of breath, showing diffuse multifocal airspace opacities consistent with acute respiratory distress syndrome

**Figure 3:**
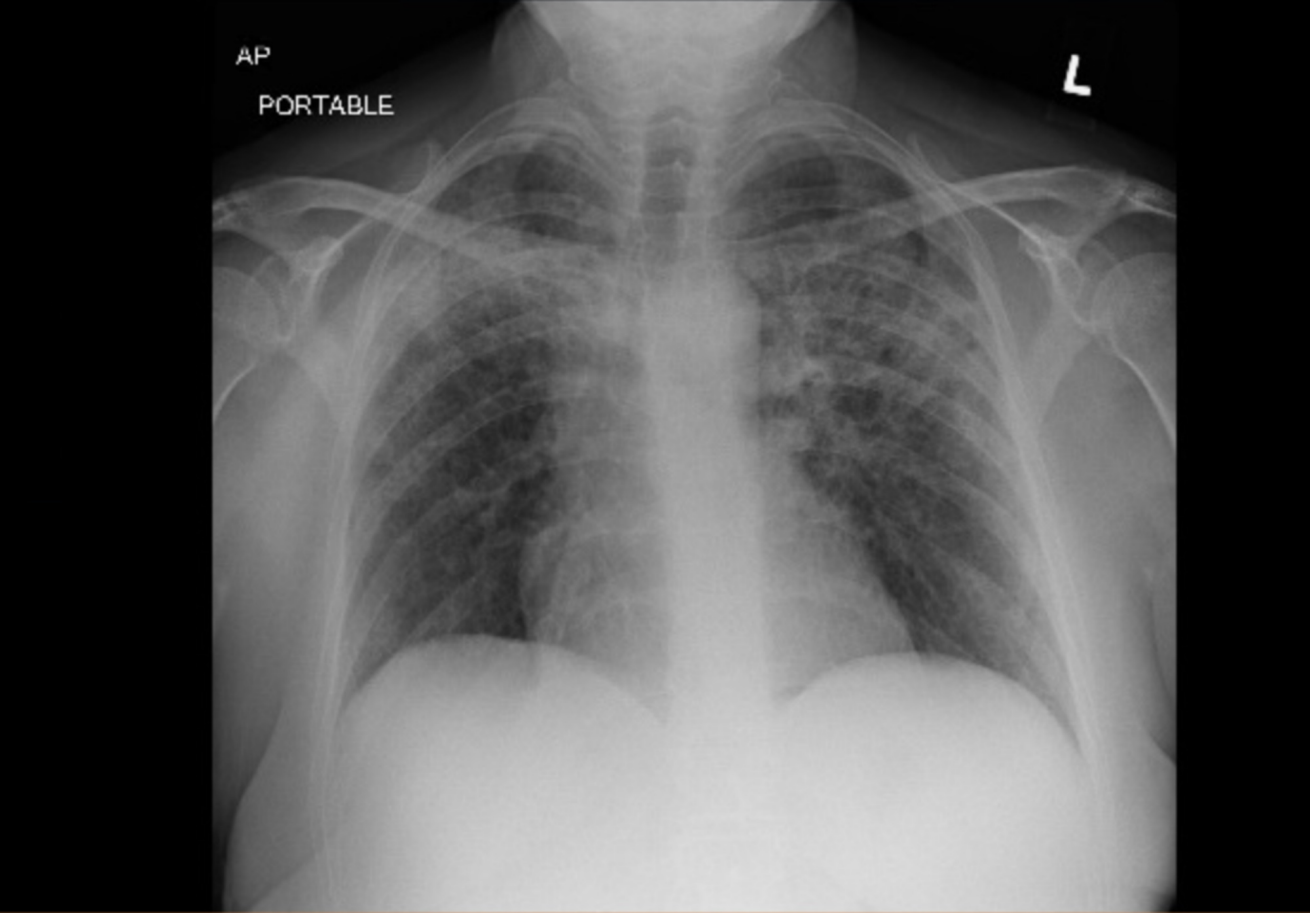
Anterior-posterior chest X-ray showing interval improvement of bilateral airspace opacities on discharge from the intensive care unit

A previously healthy, 59-year-old female was referred from Urgent Care for respiratory distress and dry cough for one week during the spring season. Outpatient chest radiographic findings were suspicious for basilar pneumonia. She admitted to a strong smoking history. She denied fever, chills, chest pain, orthopnea, sick contacts, and recent travel. Her oxygen saturation on room air was 95%, but she appeared diaphoretic and tremulous. Bilateral basilar crackles that were more prominent on the left, with mild expiratory wheezing, were heard on auscultation. Initial chest X-ray showed a small left basilar airspace opacity (Table 1). Initial blood work was within normal limits (Table 1). She was given high-dose intravenous (IV) steroids and vancomycin, levofloxacin, and piperacillin-tazobactam, and admitted for presumptive community-acquired pneumonia. In just over 24 hours of admission, she was found to be in increasing respiratory compromise. Arterial blood gas parameters were pH 7.44, pCO_2_ 37 mmHg, pO_2_ 63 mmHg, HCO_3_ 25 mmol/L, and FiO_2_ 28. Oxygen saturation dropped to 88% on 2 liters per minute via the nasal cannula. Repeat chest X-ray showed diffuse multifocal airspace opacities and the lower extremity venous duplex was negative for venous thrombosis (Figures 1-2). The patient was upgraded to the intensive care unit (ICU). The polymerase chain reaction (PCR) of the nasopharyngeal aspirate confirmed human rhinovirus (hRV). Methicillin-resistant Staphylococcus aureus (MRSA) nasopharyngeal swab, urine Legionella antigen, and sputum culture were negative. High-dose intravenous steroids were started as adjuvant therapy due to the rapid decline, presumably due to a dysregulated host immune response. Echocardiography showed a normal ejection fraction at 67%, with normal systolic and diastolic function. She was never intubated, but she was stabilized on high-flow nasal cannula oxygen therapy. She had completed a 10-day course of Levofloxacin. On discharge, repeat chest X-ray showed interval improvement in airspace opacities (Figure 3). After 10 days in the ICU, she was discharged with tiotropium and steroid taper.

**Table 1:** Initial blood profile and chemistries obtained at hospital admission

Laboratory Study	Result
White Blood Cell Count	8.67 K/uL
Hemoglobin	13.6 g/dL
Hematocrit	39.3%
Platelet Count	176 K/uL
Sodium	131 mmol/L
Potassium	3.6 mmol/L
Chloride	95 mmol/L
Carbon Dioxide	23 mmol/L
Blood Urea Nitrogen	13 mg/dL
Creatinine	0.8 mg/dL
Glomerular Filtration Rate	81 mL/min/1.73 m2
Anion Gap	13 mmol/L

## Discussion

Historically, pandemic viruses have been implicated in serious respiratory illness, such as ARDS, in immunocompetent hosts. However, the improved detection of respiratory viruses, such as hRVs, with a reverse-transcriptase polymerase chain reaction (RT-PCR) has led to increased recognition of their role in inducing a parenchymal lung injury that extends beyond that of the common cold. Among the three identified types of hRV (hRV-A, hRV-B, hRV-C), hRV-A and hRV-C cause more serious respiratory infections, including asthma exacerbations than hRV-B [2]. The exact disease mechanism is not yet elucidated, but it is known to result in diffuse alveolar damage. There may be a combination of both direct viral-mediated injury in addition to a dysregulated host immune response via cytokine-induced recruitment of inflammatory cells. There are only three reported cases of rhinovirus-induced ARDS in immunocompetent hosts on PubMed to date. These cases all affected females in early adulthood [7-9]. A study that examined the viral etiology of ARDS in Lombardy between 2009 and 2011 in 206 hospitalized patients was excluded because it is unclear if the four represented cases were from immunocompetent hosts [10]. This is the first reported case of rhinovirus-induced ARDS in a middle-aged immunocompetent adult.

The treatment of rhinovirus-induced ARDS is no different from that of any other cause. For patients who require invasive mechanical ventilation, a low-tidal-volume strategy is used (6 mL/kg of ideal body weight) to prevent the risk of barotrauma. Atelectasis and edema of injured lung tissue are less likely to be recruited by high-volume ventilation; rather, high tidal volumes are redirected to relatively normal well-aerated lung tissue, increasing the risk of hyperinflation [11]. Severe ARDS can be additionally managed using prone positioning and paralytic therapy during the first 48 hours following intubation [12]. However, less severe ARDS can be managed with non-invasive ventilation such as in this case. Bronchoscopy can be used where there is a lack of improvement or suspicion for secondary bacterial pneumonia. The use of empiric antibiotics is discouraged, as there is a risk of antibiotic resistance and a nosocomial infection while intravenous steroids produce inconsistent results [3]. The application of high-dose intravenous vitamin C was shown in the treatment of one case of rhinovirus-induced ARDS though its role is uncertain [9].

Despite studies showing rhinoviruses as one of the leading causes of severe respiratory illness requiring hospitalization, there are very few reports of hRV-induced ARDS [13]. This suggests that non-pandemic viruses are not suspected culprits. Physicians need to be more cognizant of this common respiratory pathogen, which can affect both immunocompromised and immunocompetent patients. This case highlights the need for the early identification of viral culprits, which can minimize the use of invasive diagnostic testing and antibiotic usage.

## Conclusions

hRVs should be considered a potential cause of severe ARDS. This case highlights the need for the early identification of viral culprits, which can minimize the use of invasive diagnostic testing and antibiotic usage.
